# Efficient engraftment and viral transduction of human hepatocytes in an FRG rat liver humanization model

**DOI:** 10.1038/s41598-022-18119-6

**Published:** 2022-08-18

**Authors:** Marisa Carbonaro, Jeffrey Lee, Evangelos Pefanis, Mathieu Desclaux, Kehui Wang, Alexander Pennington, Hui Huang, Alejo Mujica, Jose Rojas, Roxanne Ally, Daniel Kennedy, Michael Brown, Vitaliy Rogulin, Sven Moller-Tank, Leah Sabin, Brian Zambrowicz, Gavin Thurston, Zhe Li

**Affiliations:** Regeneron Pharmaceuticals, Inc., Tarrytown, NY USA

**Keywords:** Biological models, Liver, Hepatocytes, Genetic engineering, Rat

## Abstract

Humanized liver rodent models, in which the host liver parenchyma is repopulated by human hepatocytes, have been increasingly used for drug development and disease research. Unlike the leading humanized liver mouse model in which Fumarylacetoacetate Hydrolase (Fah), Recombination Activating Gene (Rag)-2 and Interleukin-2 Receptor Gamma (Il2rg) genes were inactivated simultaneously, generation of similar recipient rats has been challenging. Here, using Velocigene and 1-cell-embryo-targeting technologies, we generated a rat model deficient in Fah, Rag1/2 and Il2rg genes, similar to humanized liver mice. These rats were efficiently engrafted with Fah-expressing hepatocytes from rat, mouse and human. Humanized liver rats expressed human albumin and complement proteins in serum and showed a normal liver zonation pattern. Further, approaches were developed for gene delivery through viral transduction of human hepatocytes either in vivo, or in vitro prior to engraftment, providing a novel platform to study liver disease and hepatocyte-targeted therapies.

## Introduction

Due to high unmet medical needs in human liver diseases, pre-clinical models that faithfully recapitulate human liver pathology and therapeutic responses are needed. Rodent models, especially mouse and rat models, have been widely used due to their small sizes, feasibility of breeding and genetic manipulation, and availability of well characterized in-bred strains. However, rodent models seldom completely replicate the biology of human liver diseases and cannot reliably predict the effects of therapeutics (especially gene therapy agents), since there are major genomic and functional differences between rodent and human hepatocytes. Therefore, pre-clinical liver disease models that are both cost efficient and can better recapitulate human liver biology would greatly empower preclinical research related to human liver diseases and therapeutics.

Humanized liver mouse models, in which the murine liver parenchyma is repopulated by human hepatocytes, have been developed using either a uPA/SCID system (first developed by Mercer et al.^1^), an FRG system (first developed by Azuma et al.^2^), or a TK-NOG system (Hasegawa et al.^3^). Since their initial development in the early 2000s, these models have been increasingly used to model drug metabolism and toxicity, human hepatic viral infection and other diseases, and hepatocyte-targeted therapies in pre-clinical studies (for reviews see^4,5^).

Recently, efforts to generate similar chimeric rats were published^6–9^, which might overcome shortcomings of humanized liver mouse models, including small body and liver size, small total blood volume, and greater physiological differences to human. One study described an FRG rat model^9^, in which animals were genetically modified to induce hepatotoxicity and crossed into severe immuno-compromised background. This model overcame many of the limitations of earlier humanized liver rat models^6–8^ and greatly enhanced its generalization and application potential.

In this report, we independently generated a similar model through inactivation of Fumarylacetoacetate Hydrolase (Fah), Recombination Activating 1 and 2 (Rag1/2) and Interleukin 2 Receptor Subunit Gamma (Il2rg) genes in rats. Without Fah, accumulation of toxic metabolites leads to hepatoxicity in host rats, which allows for repopulation with FAH^+^ human hepatocytes. These animals could be highly engrafted with human hepatocytes from multiple sources, shown by FAH immunohistochemistry (IHC). Rats that were highly engrafted with human hepatocytes also had high serum concentrations of human albumin and complement proteins (C3 and C5). Humanized rat livers maintained proper liver zonation and could be infected with a human hepatocyte-specific adeno-associated virus (AAV). Further, we developed approaches to transduce human hepatocytes in vitro with lentivirus prior to engraftment, followed by efficient repopulation of recipient rat livers. Successful AAV infection of engrafted human hepatocytes and engraftment of lentivirus-infected primary human hepatocytes, both demonstrated here for the first time in a humanized liver rat model, will enable gene delivery/modification in humanized livers.

## Results

### Generation of FRG rat

**Figure 1:**
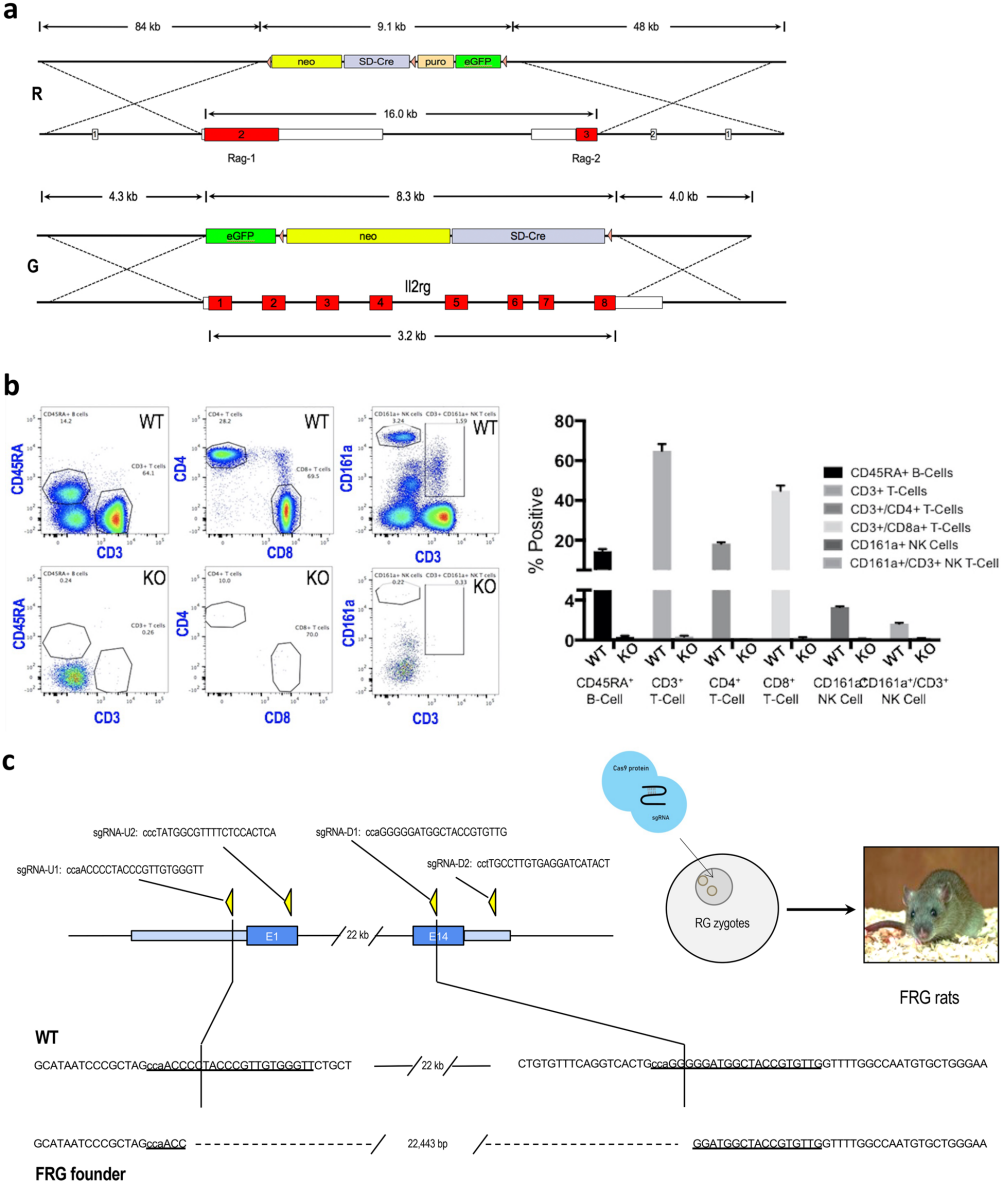
Generation of FRG rats. (**a**) Targeting vector designs for *Rag-1/Rag-2* (R) and *Il2rg* (G). The *Rag-1/Rag-2* vector deletes the entire coding sequence of both genes as well as the *Rag-1* 3′-UTR, the 5′-UTR and the intergenic region between *Rag-1* and *Rag-2*. The *Il2rg* vector deletes the entire coding sequence of Il2rg. (**b**) FACS sorting of PBMC from male RG rats. CD45RA marks B-cells. CD3, CD4 and CD8 mark T-cells. CD161a marks NK cells. RG rats lack virtually all B, T and NK cells. (**c**) *FAH* knockout and genotyping. sgRNAs were designed to cut in the 5′-UTR, exon 1, exon 14 and the 3′ UTR of the rat *FAH* gene. RNPs containing Cas9 protein and all 4 sgRNAs were electroporated into 1-cell RG embryos. F0 pups were genotyped by TaqMan and candidate FRG founders were confirmed by NGS at the *FAH* locus. A single F0 male was chosen to establish the FRG line.

We used rat embryonic stem cells (rESC) developed at Regeneron as the starting point for producing FRG rats (manuscript submitted). A plasmid-based DNA targeting vector was designed to delete the rat *Il2rg* gene by homologous recombination in rESC (Fig. 1a-G). We also designed a BAC-based DNA targeting vector to delete the linked *Rag1* and *Rag2* genes and the intergenic region between them by homologous recombination (Fig. 1a-R). Targeted rESC clones carrying the *Il2rg* or *Rag1/Rag2* mutations were identified. In separate experiments, *Il2rg* or *Rag1/Rag2* mutant rESC clones were microinjected into host blastocyst-stage rat embryos, which were then transplanted into pseudopregnant female recipients. Male chimeras born from these transfers were mated to wild-type females and, in both cases, germline transmission of the rESC genome was achieved. The *Rag1/Rag2* and *Il2rg* mutant lines were then intercrossed to produce “RG” triple knockout rats.

Immunodeficient rats harboring some combination of mutations in the *Il2rg, Rag2* and *Prkdc* genes have previously been reported^10–12^. The RG rats and parental R and G lines were characterized by FACS using markers for B-cells, T-cells and NK cells. FACS analysis confirmed decreased but detectable circulating B, T and NK in *Il2rg* KO rats. *Rag1/Rag2* KO rats also had reduced B and T cells but had increased circulating NK cells (data not shown), consistent with previous reports. RG rats had severely reduced B, T and NK cells (Fig. 1b).

Next, RG rats were mated and 1-cell embryos collected for *FAH* targeting. *FAH* mutant rats produced by CRISPR targeting in zygotes were recently described^13^. Our approach used designed sgRNAs to produce double stranded breaks in the first and last coding exons of *FAH* (Fig. 1c). This targeting strategy often results in a DNA repair event in which the chromosomal regions flanking the cut sites are ligated together, resulting in a large deletion of the region between the cut sites. We made ribonuclear protein complexes by combining recombinant Cas9 protein with the *FAH* sgRNAs and electroporated these into the RG zygotes. After transfer of the targeted embryos to surrogates, F0 animals that carried the desired *FAH* mutation were identified (Fig. 1c). The *FAH* mutation was bred to homozygosity to produce FRG rats. Using RG zygotes rather than WT for *FAH* targeting bypassed many months of breeding, thus reducing the number of animals used to produce the line.

### Engraftment of wildtype rat hepatocytes rescued lethal phenotype of FRG rats

The protein product of the Fah gene, fumarylacetoacetate hydrolase, is a critical enzyme in the tyrosine metabolic pathway that catalyzes the degradation of the intermediate metabolite fumarylacetoacetate (FAA) into fumarate and acetoacetate. Inactivation of Fah results in accumulation of FAA, affecting the liver and kidney where Fah is mainly expressed, and results in a lethal phenotype. Fah KO animals can be maintained by treatment with 2-(2-Nitro-4-trifluoromethylbenzoyl)-1,3-cyclohexanedione (NTBC), an inhibitor of 4-hydroxyphenylpyruvate dioxygenase (HPPD), which acts by blocking the accumulation of toxic metabolites (Reviewed by Grompe^14^).

**Figure 2:**
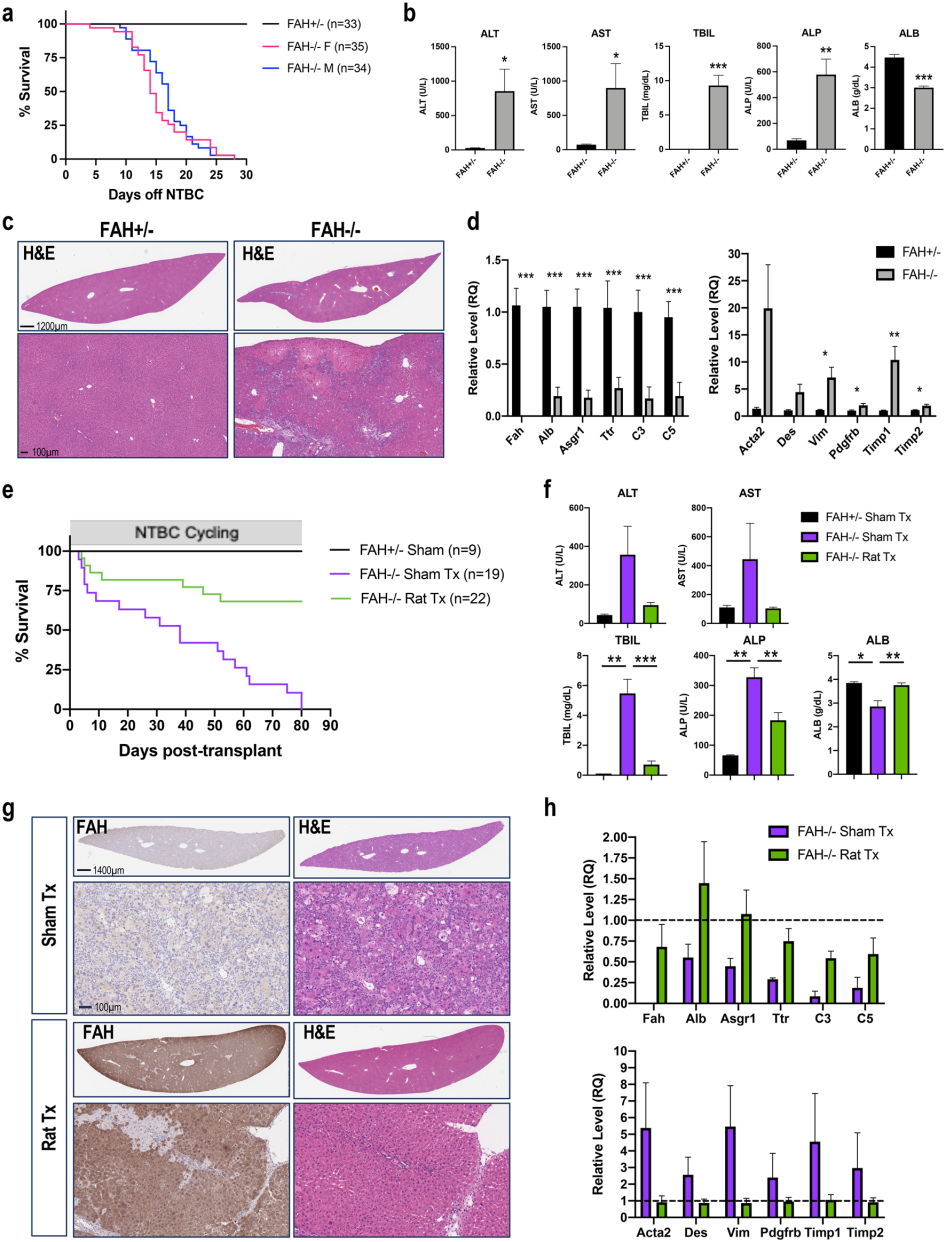
Engraftment of wildtype rat hepatocytes rescues lethal phenotype of FRG rats. (**a**) Kaplan–Meier survival curves of male and female Fah^+/−^ (n = 33) and Fah^−/−^ (n = 35F, 34M) rats after NTBC removal. (**b**) Serum ALT, AST, TBIL, ALP and ALB from terminal bleeds from 10 Fah^+/−^ and 13 Fah^−/−^ rats at day 14–20 after NTBC withdrawal. (**c**) H&E staining from one representative Fah^+/−^ and Fah^−/−^ showing extensive liver damage with NTBC withdrawal. (**d**) TaqMan real-time PCR data showing a decrease in hepatocyte-expressed genes (left) and an increase in fibrogenesis-related genes (right) in Fah^−/−^ vs. Fah^+/−^ rats. (**e**) Survival curve of Fah^+/−^ (n = 9) rats or *Fah*^−/−^, with (n = 22) or without (n = 19) a transplant of wild-type rat hepatocytes, while following an NTBC cycling schedule. (**f**) Serum ALT, AST, TBIL, ALP and ALB levels of rats after sham surgery vs. rat hepatocyte transplant. (**g**) H&E and FAH IHC showing repopulation of the FRG rat liver with wild-type rat hepatocytes. (**h**) TaqMan data showing a rescue of gene changes in Fah^−/−^ rats repopulated with wild-type hepatocytes vs. Fah^−/−^ rats that received sham surgery, relative to Fah^+/−^ rats with no liver damage (dotted line). Data in (**b**), (**d**) and (**f**) shown as mean ± SEM. *p < 0.05, **p < 0.01, ***p < 0.001, Unpaired *t* test.

Earlier studies showed that Fah KO rats died within 6 weeks of NTBC withdrawal due to liver and kidney damage^13^. Consistently, the FRG triple knockout rats also needed NTBC to survive. To evaluate the suitability of FRG rats as recipient animals for hepatocyte engraftment, as proven in FRG mice, we assessed whether repopulation of the FRG rat liver parenchyma with wildtype rat hepatocytes carrying an intact Fah gene would allow for survival without NTBC. Without transplantation of wildtype hepatocytes, both male and female rats died within 4 weeks of NTBC withdrawal (Fig. 2a), which was more rapid than the death of Fah KO rats reported previously^13^. Serum chemistry showed a sharp elevation of AST, ALT, TBIL and ALP, as well as decreased ALB levels in FRG rats 14–20 days after NTBC withdrawal (Fig. 2b). Further, liver sections showed severe tissue damage marked by extensive hepatocyte necrosis, bile duct hyperplasia with lymphocyte infiltration, as well as multifocal hemorrhages (Fig. 2c). Accordingly, TaqMan real-time PCR analysis of the livers of Fah KO animals showed a significant down-regulation of hepatocyte-expressed genes including Alb, Asgr1, Ttr, C3 and C5, as well as upregulation of genes involved in fibrogenesis, such as Acta2, Des, Vim, Pdgfrb, Timp1 and Timp2 (Fig. 2d). In contrast, no lethality or liver damage was observed in control animals with heterozygous Fah alleles (Fig. 2a–c). Further, the surgical procedure for implantation accelerated the death of FRG rats, leading to a less-than-2-week survival after NTBC withdrawal, and implantation of wildtype rat hepatocytes failed to prevent the rapid death in the complete absence of NTBC (Supplementary Fig. 1).

We next applied an NTBC on/off cycling scheme to reduce the liver damage, and allow for the repopulation of Fah null livers with wildtype hepatocytes. As shown in Fig. 2e, with NTBC cycling, 65% of FRG rats that received sham surgery died by day 50, and 100% of animals died by day 80 post-surgery. In comparison, survival was significantly improved after implantation of wildtype rat hepatocytes into FRG rat livers, and 70% of animals implanted with FAH^+^ hepatocytes survived beyond 80 days post-surgery. Accordingly, serum chemistry showed that abnormalities in multiple indicators of liver function in FRG rats after sham surgery, including elevated ALT, AST, ALP, TBIL and decreased ALB, were reversed by engraftment of wildtype hepatocytes (Fig. 2f). The livers of these animals showed a high degree of repopulation by FAH^+^ donor hepatocytes and improved morphology (Fig. 2g). Consistently, TaqMan analysis confirmed that abnormalities in gene expression in FRG rat livers were also corrected by wildtype hepatocyte engraftment (Fig. 2h).

### Cross species engraftment of donor hepatocytes and liver humanization in FRG rats

**Figure 3:**
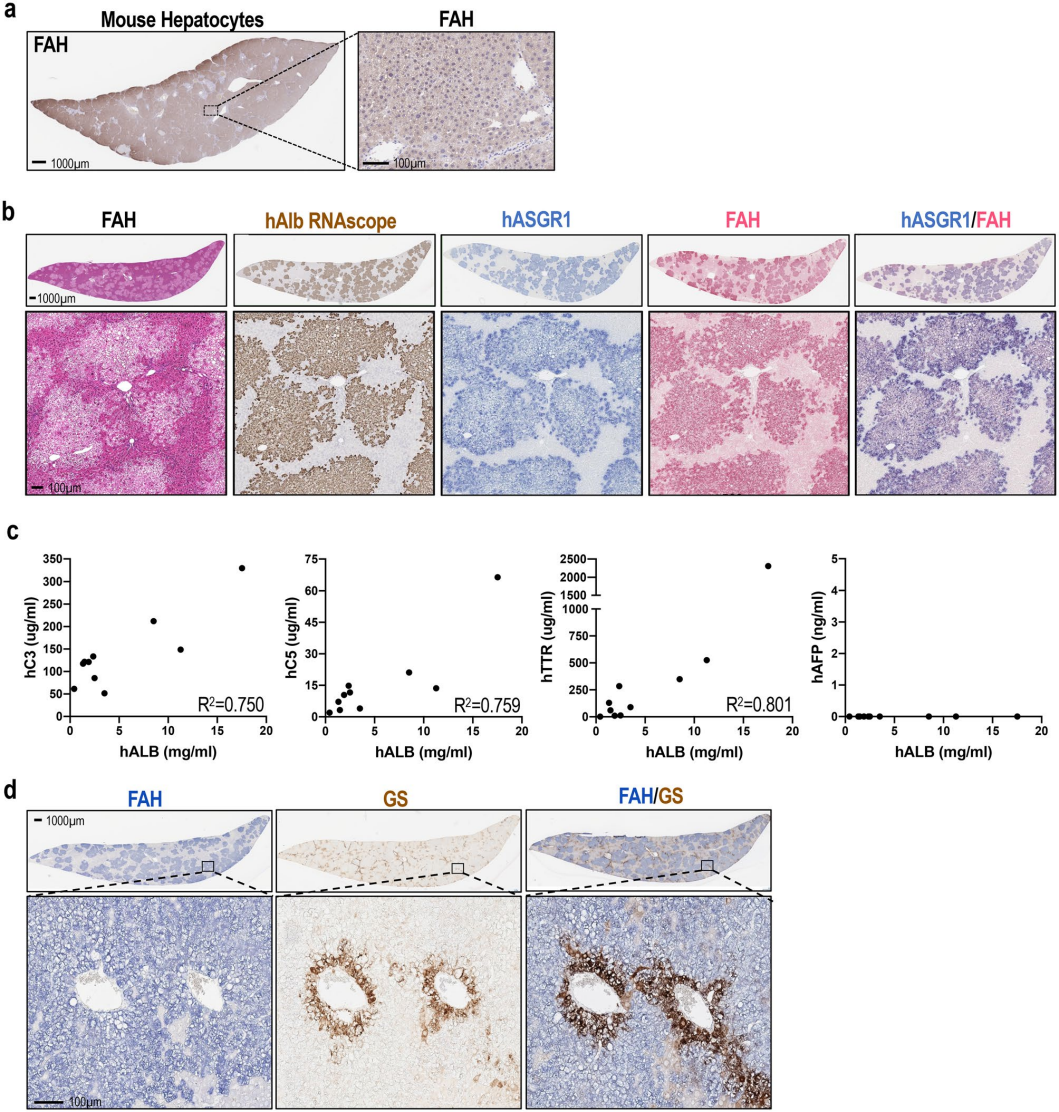
Cross species engraftment of donor hepatocytes and liver humanization in FRG rats. (**a**) Engraftment of murine hepatocytes in FRG rats, shown by FAH IHC. (**b**) A highly engrafted humanized liver rat showing pale H&E staining characteristic of engrafted human hepatocytes, and confirmed by positive human albumin RNAscope staining and colocalization of FAH and human ASGR1 IHC staining. (**c**) Correlation of human albumin (mg/ml), human complement proteins, C3 and C5 (μg/ml), human TTR (μg/ml) and human AFP (ng/ml) in the serum of rats engrafted with primary human hepatocytes. (**d**) GS staining in FAH-positive hepatocytes around the central vein confirms that humanized livers maintain proper liver zonation.

After showing that engraftment of FAH^+^ rat hepatocytes could significantly improve the survival of FRG rats, we next assessed whether the immuno-suppressive environment of these rats would allow engraftment of FAH^+^ hepatocytes from other species. To this end, a similar implantation procedure and NTBC cycling scheme were applied on FRG rats following engraftment of murine and human hepatocytes. As shown in Fig. 3a,b, cross-species engraftment was confirmed by detection of abundant Fah^+^ cells in the livers of both murine and human hepatocyte-implanted FRG rats. The hepatocyte specification of engrafted human cells in this model was confirmed by colocalization of human albumin (hALB), human Asialoglycoprotein Receptor 1 (hASGR1) and FAH through RNAscope and IHC (Fig. 3b). Consistently, increased murine or human albumin levels in host rat serum correlated to higher repopulation rates of donor hepatocytes, as estimated by abundance of FAH^+^ cells in engrafted liver sections (Supplemental Fig. 2b,d). As shown by FAH IHC, human hepatocyte engraftment resulted in clusters of FAH^+^ cells in the liver, suggesting clonal expansion (Fig. 3b). Further, we showed high engraftment of both freshly thawed and cultured primary human hepatocytes, from multiple donors, into FRG rat livers (Supplemental Fig. 2e). In addition to human albumin, engraftment of human hepatocytes also led to the presence of hepatocyte-derived human proteins such as complement components 3 and 5 (C3/C5), as well as Transthyretin (TTR) in the serum of host rats. As expected, increased expression of all 3 of these markers associated with elevated serum human albumin levels (Fig. 3c). In contrast, human alpha-fetoprotein (AFP), which is normally expressed by fetal hepatocytes or liver tumors but not normal mature hepatocytes was not detected in the serum of humanized liver rats (Fig. 3c). Further, pericentral staining of glutamate-ammonia ligase [also called glutamine synthetase (GS)] in humanized regions suggested the maintenance of proper metabolic zonation of human hepatocytes (Fig. 3d).

### Human-specific gene delivery in humanized liver rats through viral infection

**Figure 4:**
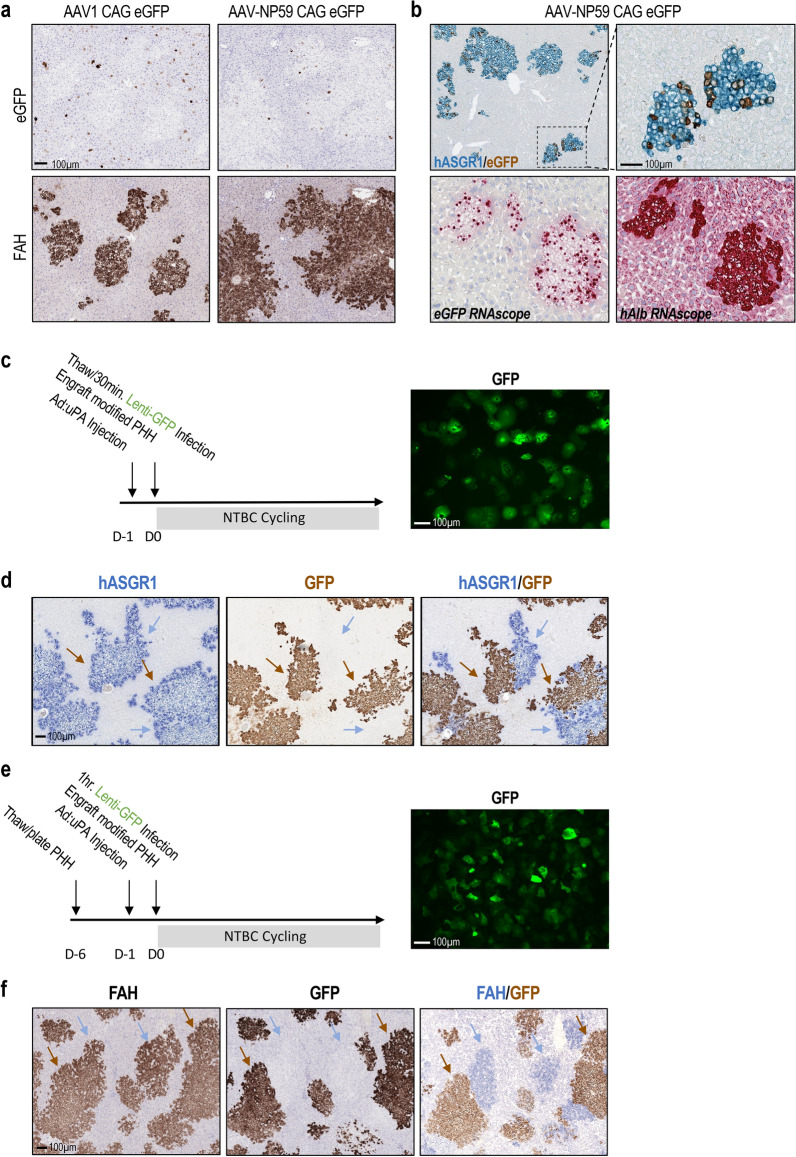
Human-hepatocyte-specific gene delivery in humanized liver rats. (**a**) eGFP and FAH IHC show different staining patterns after AAV1 or AAV-NP59 mediated eGFP delivery to humanized livers. (**b**) AAV-NP59 shows preference for human hepatocytes, confirmed by double IHC for hASGR1 (blue) and eGFP (brown) in top panels, and eGFP or human albumin RNAscope (bottom panels). (**c**) Experiment timeline and pre-engraftment GFP expression in human hepatocytes (donor FCL) transduced with Lenti-GFP for 30 min at 5.00E + 04 viral genomes (VG)/cell in suspension. (**d**) hASGR1 and GFP single or double IHC in rat livers engrafted with lentivirus-infected, freshly-thawed human hepatocytes. (**e**) Pre-engraftment GFP expression in human hepatocytes (donor FCL) after 6 days in culture and a 1-hr infection with Lenti-GFP at 5.00E + 04 VG/cell. (**f**) FAH and GFP IHC staining confirms engraftment of lentivirus infected, cultured hepatocytes. Brown arrows point to GFP^+^ human hepatocytes. Blue arrows mark GFP^−^ human hepatocytes.

In order to create models of liver disease, we next attempted to perform gene delivery into human hepatocytes. For in vivo human-specific gene delivery in humanized liver rats, we examined AAV-NP59, an AAV serotype that has been shown to transduce human hepatocytes in humanized liver mice^15^. In this study, an AAV vector encoding an eGFP reporter gene was packaged into either AAV1 or AAV-NP59, and the virus was introduced into humanized liver rats through IV injection. As shown in Fig. 4a, in the livers of AAV1 treated animals, eGFP positive cells were only observed in FAH negative regions (i.e., host rat hepatocytes). In comparison, clusters of GFP/hASGR1 double-positive cells were detected in the livers of rats treated with AAV-NP59-eGFP, demonstrating successful transduction of human hepatocytes. In these rats, no eGFP expression was detected in hASGR1 negative regions, confirming human-hepatocyte specific transduction.

Despite human specificity, only a small fraction of human hepatocytes were positive for eGFP expression by IHC (Fig. 4b, upper panel). To determine whether the low frequency of eGFP expression reflects low transduction efficiency, we performed in-situ hybridization using a probe that detects both mRNA and DNA sequence of the eGFP gene delivered by the virus. As shown in Fig. 4b (lower panel), we observed positive eGFP ISH signal in the nuclei of most, if not all, human hepatocytes. However, cytoplasmic eGFP ISH signal was limited to only a subset of hepatocytes, consistent with the low frequency of eGFP protein expression. This observation suggests that AAV genomes are delivered effectively to engrafted human hepatocytes by AAV-NP59, but active transcription of the AAV-delivered transgene is limited to a subset of cells.

As an alternative approach to deliver transgenes into human hepatocytes, we also tested ex vivo transduction with lentiviral vectors. To this end, freshly thawed donor human hepatocytes were incubated with a lentiviral vector expressing eGFP, followed by immediate implantation into FRG rats. Successful hepatocyte transduction was confirmed by detection of eGFP through florescence microscopy in cultured cells at 4 days post-infection (Fig. 4c). We estimated a transduction efficiency of ~ 50% by comparing florescent and light microscopic fields (data not shown). The engrafted rats showed a steady increase of human albumin expression starting at 10–12 weeks post-implantation, indicating successful repopulation of the host rat liver with lentivirus-treated human hepatocytes (Supplementary Fig. 3a).

Immunohistochemistry on the liver of an FRG rat 16 weeks post-implantation with lentivirus-infected human hepatocytes showed the presence of many hASGR1 positive patches, confirming the successful engraftment of donor cells (Fig. 4d). In these studies, about 30–50% of the hASGR1 positive cells were also eGFP positive, confirming successful engraftment of lentivirus transduced human hepatocytes. As shown in Fig. 4d, we also observed hASGR1 positive regions that were eGFP negative, suggesting the co-engraftment of non-transduced hepatocytes, or transduced hepatocytes with silenced transgenes. Unlike the intermingled pattern of eGFP positive and negative human hepatocytes observed in AAV-NP59 infected livers, engraftment of lentivirus infected human hepatocytes showed clear segregation between eGFP positive and negative cells, suggesting expansion of both transgene-expressing and non-expressing clones.

While it was encouraging to be able to engraft lentivirus-infected, freshly thawed primary human hepatocytes and maintain expression of the transgene in rat livers, the short time window between thawing and implantation of donor hepatocytes could limit the application of this technique. Therefore, we attempted to perform gene delivery into cultured hepatocytes before engraftment into FRG rats. To this end, cultured human hepatocytes were incubated with the lentiviral vector expressing eGFP for 1 hour before being dissociated and implanted into FRG rats (Fig. 4e). Again, increasing human albumin was detected in the serum of engrafted animals (Supplementary Fig. 3b), and repopulation of host rat livers with human hepatocytes was verified by positive FAH IHC (Fig. 4f). Importantly, clusters of eGFP positive cells were colocalized with FAH expression (Fig. 4f), confirming engraftment of lentivirus-infected human hepatocytes. Similar to observations from humanized liver rats engrafted with freshly thawed hepatocytes (Fig. 4d), segregated GFP^+^/FAH^+^ and GFP^−^/FAH^+^ cell clusters were also detected in livers engrafted with cultured human hepatocytes (Fig. 4f). Thus, both techniques allow lentiviral manipulation of human hepatocytes in vitro prior to engraftment into FRG rats.

## Discussion

The success of the FRG humanized liver mouse model has led to demand for an equivalent recipient rat model. However, despite the successful generation of Fah null and Rag2 null, and Il2rg null rats by different groups more than 5 years ago^10,13,16^, bringing all 3 alleles together in rats through crossing is time consuming and logistically challenging, and the first FRG rat model was just published recently^9^. In this report, we sequentially introduced additional mutations through gene targeting in one-cell-stage embryos of animals carrying the homozygous alleles already modified. With this strategy, we were able to rapidly generate FRG rats without large scaled-up breeding schemes. A similar approach could also be used to perform multi-allele gene targeting in rats without technically challenging serial targeting in ES cells, or resource consuming breeding programs.

Compared to other published humanized liver rat models, the FRG rat models independently generated by us and others^9^ have several advantages. First, simultaneous inactivation of Rag1/2 and Il2rg genes makes the recipient rats highly immune-permissive. No radiation^6^ or administration of immune suppressive reagents^7,8^, as done by others, was required to avoid immune-rejection of xenografted hepatocytes. Intra-species engraftment with non-syngeneic wildtype donor rat hepatocytes, and cross-species engraftment of both murine and human hepatocytes was successful in FRG rats. In addition, the liver damage caused by Fah-KO-induced FAA accumulation in host rat hepatocytes was controlled by NTBC administration^13,17^. Therefore, Fah deletion and NTBC treatment not only removed the requirement for highly invasive surgical procedures such as hepatectomy, but also provided flexibility for the timing of engraftment procedures. Further, the NTBC on-and-off cycling schedule can be adjusted to allow the appropriate balance between damage of host hepatocytes and repopulation of donor hepatocytes to ensure animal survival and maximize engraftment of donor hepatocytes^2^.

A recent study^9^ described a similar model of humanized rat livers, with several potentially relevant technical differences. First, although NTBC on-and-off cycling (albeit with slightly different schedule) was applied after implantation in both the current study and the other FRG rat humanized liver model described by Zhang et al.^9^, we pre-conditioned recipient rats through dosing with adenovirus encoding urokinase-plasminogen activator (uPA) 24 hours before hepatocyte implantation, rather than treating with retrosine 2 weeks before cell transplantation followed by gradual NTBC withdrawal. In addition, the approaches to genetically alter the rat ES cells were somewhat different. In particular, both Rag1 and Rag2 genes were inactivated in our approach, rather than just Rag2 gene. Further, in addition to using donor liver cells freshly thawed from cryo-preservation as described in the other report^9^, we also successfully engrafted cultured human hepatocytes.

Successful liver humanization in rats leads to the possibility of modeling human liver diseases in these animals. Therefore, we made first-in-the-class attempts on human-specific gene delivery and genetic targeting of hepatocytes in humanized liver rat model. To this end, we expressed the reporter transgene, eGFP, in humanized livers, through either AAV or lentivirus-mediated gene delivery. A human-specific engineered serotype AAV-NP59 was shown to be able to transduce and deliver transgenes into humanized liver rats in vivo for the first time. However, despite the presence of AAV genomes in the majority of human hepatocytes in AAV-NP59 infected rats, only a subset expressed eGFP protein. While the mechanism driving the disconnect between gene delivery and gene expression is unclear, some potential hypotheses include transcriptional repression of AAV episomes, perhaps through epigenetic modifications, or the accumulation of non-functional nuclear genomes due to a non-productive infection pathway. In addition, since the AAV genome does not naturally integrate into the hepatocyte genome, the vector genomes will be diluted during division or proliferation of human hepatocytes. Therefore, AAV-mediated in vivo gene delivery in humanized livers may be most suitable for creating “hit-and-run” gene modification events in a subset of human hepatocytes within humanized livers, such as introducing sporadic somatic mutations through CRISPR/Cas9-mediated gene editing.

We further demonstrated an orthogonal method of transgene delivery to human hepatocytes in humanized liver rats, through lentivirus infection of donor hepatocytes prior to engraftment. Here, we showed that lentivirus can efficiently transduce donor primary human hepatocytes, not only in suspension upon thawing from cryopreservation, but also in culture after days of ex-vivo culturing. While high transduction efficiency of donor hepatocytes was reached prior to implantation, both infected and uninfected hepatocytes repopulate recipient rat livers successfully. To repopulate rat livers with only transduced cells, appropriate purification procedures such as drug selection, flow cytometry or cell exclusion columns might be needed before engraftment. Nevertheless, highly efficient lentiviral transduction of donor hepatocytes and robust transgene expression in human engraftments makes it feasible to ectopically express transgenes or perform targeted editing of endogenous genes in humanized livers. Taken together, lentiviral or AAV-mediated gene delivery to human hepatocytes, alone or combined, can provide countless new opportunities for utilizing humanized liver rats in human disease and therapeutic modeling. In addition to conventional applications of humanized liver animal models in basic research or pre-clinical drug development, such as modeling human hepatic pathogen infection, drug metabolism studies, and AAV or retroviral mediated gene therapy^5^, human hepatocyte-specific gene delivery described in this report would allow modeling genetic liver diseases humanized liver animals with human hepatocytes carrying the pathogenic genetic alterations.

## Materials and methods

### Animals

All animal studies were approved by the the Institutional Animal Care and Use Committee (IACUC) guidelines at Regeneron Pharmaceuticals, Inc and performed in accordance with “*IACUC Regulations and Processes Governing the Use of Animals In Research at Regeneron*”. Animals were maintained with 2-(2-Nitro-4-trifluoromethylbenzoyl)-1,3-cyclohexanedione (NTBC)-containing drinking water at a concentration of 16 mg/l. NTBC was purchased from Medinoah (90-1596). The drinking water also contained antibiotics, Sulfamethoxazole (640 μg/ml, RPI S47000) and Trimethoprim (128 μg/ml, RPI T59000), in 3% dextrose water.

### Production of targeting vectors for rats

The Il2rg targeting vector was constructed by PCR-amplifying 5 kb homology arms that flank the Il2rg locus, using Dark Agouti genomic DNA. 5′ and 3′ homology arms were ligated to a selection cassette and an origin of replication (R6K) to create the final targeting vector. The Rag1/Rag2 Large Targeting Vector (LTVEC) was constructed as described before^18^ using a rat BAC library that was constructed using genomic DNA from DA rat ES Cells (Lucigen Corporation, Middleton, WI). BACs containing the Rag1/Rag2 loci were targeted with a plasmid containing a drug selection cassette flanked by 100 bp of synthetic rat sequence homologous to the target region. Rat sequences were taken from the public Reference Sequence (Rnor_6.0). The final LTVEC was assembled by bacterial homologous recombination.

### Production of targeted rESC-derived rats

rESC were passaged 48 h before electroporation and cultured in 2i media^19^. rESC colonies were collected, dissociated and counted. 4 × 10^6^ cells were electroporated with 2 μg of vector using a BTX ECM 630 electroporation system (BTX, Holliston MA). After electroporation the cells were plated overnight in 2i media, followed by 10 days of puromycin resistance selection (0.75 mg/ml). After selection, individual colonies were expanded into individual wells of a 96-well plate. Screening for targeted rES cell clones was done using TaqMan (Applied Biosystems) qPCR assays in a Thermo Fisher ViiA 7 Real-Time PCR System to determine the CT for each reaction (the point in the PCR at which the fluorescence signal reaches a preset threshold). CT was used to determine loss-of-native-allele by counting a reduction in intact copies of the target allele compared with copies of a reference gene (Valenzuela, 2003). For rESC microinjection, ~ 15 rESC each were microinjected into the blastocoels of Sprague Dawley (SD) blastocysts, followed by implantation of 12–14 injected blastocysts into the uteri of 8 week-old pseudopregnant SD female rats. Chimeric F0 rats were identified by coat color chimerism; 10 week-old male chimeras were mated to SD female rats to produce F1 litters. Agouti coat color in F1 pups indicates germline transmission of the rESC genome; these animals were genotyped by TaqMan to confirm the presence of the targeted allele. Targeted F1 male rats were then mated to establish the Il2rg and Rag1-Rag2 KO lines, which were then bred together to establish the RG line.

### Production of FAH-targeted rats

CRISPR sgRNAs were designed to target the first and last coding exons of the rat FAH gene. Female Il2rg/Rag1-Rag2 (RG) triple KO rats were superovulated by PMSG/hCG injection and mated overnight with male RG rats. Fertilized 1-cell embryos were collected the following morning for CRISPR electroporation. 2.5 μg of each sgRNA were mixed together and incubated with 20 μg Cas9 protein for 15 min at room temperature. RNPs were electroporated into the RG zygotes, which were subsequently transferred to the oviducts of pseudopregnant SD females. The dams were administered NTBC-containing drinking water at 16 mg/ml prior to litter drop and through F1 weaning. All F1 pups were genotyped for targeted collapse of the FAH gene as well as to confirm the RG mutations. Male F1 rats harboring all the targeted mutations (FRG rats) were mated to establish the line. FRG rats were maintained on NTBC water.

### Hepatocyte transplantation

Cryopreserved rat and mouse hepatocytes were purchased from ThermoFisher (RTCP10) and Yecuris (20-0019), respectively. Human primary hepatocytes were purchased from BioIVT (F00995-P and M00995-P, cryopreserved) and Pheonix Bio (PXB-cells^®^, pre-plated). An adenoviral vector expressing human urokinase (uPA) was purchased from Yecuris (CuRx™ uPA Liver Tx Enhancer, 20-0029), and injected by tail vein I.V. injection, 24 h prior to hepatocyte transplant at 1.25e10 pfu/250 g body weight. Hepatocyte number and viability was determined by ViaStain AOPI Staining Solution (Nexcelom CS2-0106), using the Nexcelom Cellometer Auto 2000. 5–10 million live hepatocytes in 500 μl Roswell Park Memorial Institute (RPMI) 1640 Medium were injected intrasplenically via a 32 gauge needle. NTBC was withdrawn from the drinking water on the day of transplant to initiate NTBC on-and-off cycling scheme. NTBC was cycled with 4-day-off/3-day-on schedules for 2 weeks, 7-day-off/3-day-on for 4 weeks followed by 11-day-off/3-day-on schedules until the animals sacrificed. Body weights of the animals were closely monitored and any animals that lost over 15% of bodyweight during an NTBC-off period were put back on NTBC until the initiation of next NTBC-off period of the rest of the cohort.

### ELISAs and serum biochemical analysis

Starting at 6 weeks post-transplant, hepatocyte engraftment was monitored by measuring several hepatocyte-produced proteins in the serum. Blood was collected from the submandibular vein and spun in MiniCollect serum separator tubes (#450472). After a 1:10,000 or 1:100,000 dilution, human or mouse albumin was measured in the serum of engrafted rats using either the Human Albumin ELISA Kit (abcam, ab108788) or Mouse Albumin ELISA Kit (abcam, ab207620), following the manufacturer’s protocol. Additional human proteins were measured in the serum using the Human alpha Fetoprotein (ab108838), Human Transthyretin (ab108895), Human Complement C3 (ab108822) or C5 (ab125963) ELISA Kits, following the manufacturer’s guidelines. For serum chemistry, levels of Aspartate Aminotransferase (AST), Alanine Aminotransferase (ALT), Albumin (ALB), Total Bilirubin (TBIL), Alkaline Phosphatase (ALP), were measured using ADVIA assays.

### Histology, immunohistochemistry, and RNAscope

Livers and kidneys were fixed in 10% normal buffered formalin for 24 h, washed and stored in 70% ethanol until paraffin embedding and sectioning. Sections were stained by hematoxylin and eosin (H&E) and Picrosirius Red, according to standard protocols (staining performed by Histoserv, Inc.). Engrafted hepatocytes were detected by IHC staining for FAH (ab151998) and human ASGR1 (ab254261), or RNAscope staining for human albumin (ACD, 457511). Liver zonation was observed by IHC for GS (ab125724), and viral transduction was monitored by GFP, by both IHC (ab183734) and RNAscope (EGFP probe, ACD 400281). All histology slides were scanned using a Leica Aperio AT2 scanner. FAH IHC staining was quantified using ImageJ.

### Hepatocyte culture

Primary human hepatocytes are thawed in Cryopreserved Hepatocyte Recovery Medium (CHRM, CM7000, ThermoFisher) and plated using Primary Hepatocyte Thawing/Plating supplements (CM3000, ThermoFisher), with a 1:40 dilution of Matrigel (354277, Corning) and at least 3% fetal bovine serum (FBS). 24 h post-plating, media is changed and Primary Hepatocyte Maintenence Supplements (CM4000, ThermoFisher), plus 1% FBS are added. All supplements are added to Williams E Media, with no phenol red (A1217601, ThermoFisher).

### Lentiviral vectors production and titration

Lentiviral particles were produced following standard lipofectamine-mediated co-transfection of HEK 293T cells with the transfer plasmid encoding eGFP under the CMV promoter (pLVX-CMV-EGFP, subcloned from pLVX-EF1a-IRES-puro plasmid, Takara), a second generation packaging plasmid encoding the gag, pol and rev genes (psPAX2, obtained from the Tronolab at Ecole Polytechnique Fédérale de Lausanne, Switzerland) and a plasmid encoding the vesicular stomatitis virus envelope glycoprotein G (VSV-G) as envelope plasmid (pMD2-G, Tronolab).The day before transfection cells were washed with phosphate buffered saline solution (PBS) once then detached from vessel with TrypLE™ Express (Life Technologies). After neutralization of TrypLE Express with cell medium containing FBS, cells were centrifuged at 1200 rpm for 5 min at 25 °C, then resuspended in complete DMEM medium, counted and seeded in 150 mm cell culture dishes at a density of 10 × 10^6^ cells/plate. On the day of transfection, the cell culture medium was replaced by fresh Opti-MEM medium (Gibco/Life Technologies) supplemented with 25 nM chloroquine (Sigma-Aldrich). The DNA mix was prepared by mixing 20 μg of transfer plasmid DNA, 20 μg of packaging plasmid and 10 μg of envelope plasmid, 1.5 ml of Opti-MEM with 60 μl of PLUS™ Reagent (Life Technologies). In parallel 100 μl of lipofectamine^®^ TLX (Life Technologies) was diluted in 1.5 ml of OptiMEM medium. DNA mix was then added to the lipofectamine mix and the new combined solution was incubated at room temperature for 20 min before being added directly to the cells dropwise. The culture medium was changed 6–8 h after transfection and the cells were then incubated for 48 h at 37 °C in an incubator with 5% CO_2_ atmosphere. At day 2 post- transfection, cell media containing the lentiviral particles were centrifuged for 10 min at 3000 rpm to remove the debris, then passed through a 0.45 um pore size filter. The filtered supernatants were then treated with 1 µg/ml DNAse and 1 mM MgCl_2_ for 15 min at 37 °C to remove residual DNA. For concentrating the lentiviral vectors batch, the supernatants were then ultracentrifuged at 27,100 rpm for 90 min. After ultracentrifugation, pellets were resuspended in a suitable volume of PBS (50–100 μl) overnight. The resuspended virus was finally processed through a serie of short centrifugations (30 s at 13,500 rpm) to clarify the lentiviral solution of remaining debris. The batches of lentiviral particles were titrated by RT-qPCR using a SYBR^®^ technology-based kit from Clontech/Takara then stocked at − 80 °C until use for transduction.

### Adeno-associated virus (AAV) production

Recombinant AAV was produced by transient transfection of HEK 293T cells. Briefly, cells were transfected with AAV Rep-Cap, Adenovirus Helper, and AAV genome plasmids using PEI-Max (Polysciences). Virus containing supernatant were concentrated by tangential flow filtration and cells were lysed by sequential freeze and thaw (3 ×). Lysates were treated with Benzonase (Millipore Sigma) for one hour at 37 °C and clarified by centrifugation and filtration (0.2 µm PES). AAV was purified from clarified cell lysates and concentrated supernatant by iodixanol gradient ultracentrifugation as previously described (Source below). Virus fractions were concentrated and buffer-exchanged to 1 ×PBS + 0.001% Pluronic F68 (Thermo Fisher Scientific) using Amicon 100 kDa MWCO Ultra Centrifugal filters (Millipore Sigma). AAV genomes were quantified by qPCR using TaqMan primers and probes specific for inverted terminal repeats. A standard curve was generated using serial dilutions of virus with a known concentration.

### Lentivirus infection and AAV delivery

AAVs were delivered intravenously by tail-vein injection at 5.00E + 11–1.00E + 12 viral genomes (VG)/rat. Lentivirus infection of human hepatocytes was done *ex-vivo* prior to hepatocyte engraftment, at an MOI of 5.00E + 04 VG/cell. Cells were infected for 30 min–1 h, either in suspension immediately upon thawing cryopreserved cells, or while in culture, washed with PBS, and prepared for engraftment as above. Fluorescent GFP images were taken on a Nikon Eclipse Ti-S Inverted Microscope (40 ms exposure).

### TaqMan real-time polymerase chain reaction (PCR)

Tissues were homogenized in RNALater and purified using MagMAX-96 for Microarrays Total RNA Isolation Kit (Ambion by Life Technologies). Genomic DNA was removed using RNase-Free DNase Set (Qiagen). mRNA was reverse-transcribed into cDNA using SuperScript VILO Master Mix (Invitrogen Life Technologies) and Veriti 96-well PCR Thermal Cycler (Thermo Fisher Scientific). cDNA was amplified with SensiFAST Probe Hi-ROX (Meridian Life Science) using 12k Flex System (Applied Biosystems). PCR reactions were done in triplicate. GAPDH was used to normalize cDNA input differences. Data reported as comparative CT method using delta delta CT. Probes listed in the Supplementary Materials.

## Supplementary Information

Supplementary Information.

## Data Availability

The authors confirm that the study is reported in accordance with ARRIVE guidelines. All data generated or analysed during this study are included in this published article and its supplementary information files. Any additional data that support the findings of this study are available from the corresponding author on reasonable request.
