# Supplementary material for: Efficient engraftment and viral transduction of human hepatocytes in an FRG rat liver humanization model
Source: Sci Rep. 2022 Aug 18;12:14079. doi: 10.1038/s41598-022-18119-6 (PMC9388686; doi:10.1038/s41598-022-18119-6)
Supplement: Supplementary file 1 — Supplementary Information. [file 41598_2022_18119_MOESM1_ESM.pdf]

Supplementary Figure 1. Implantation of Wildtype Rat Hepatocytes Failed to Prevent The Rapid Death In The Complete Absence Of NTBC

A. Kaplan-Meier survival curves of Fah$^{+/-}$ (Het: Sham, n = 7) and Fah$^{-/-}$ rats after sham surgery (KO: Sham, n=7) or transplant of WT rat hepatocytes (KO: Rat, n = 8) rats, after complete NTBC removal. 7 out of 8 of the implanted animals died by day 10. NTBC cycling was applied between day 10 and day 50 to the only one animal survived. **B.** FAH IHC shows engraftment of scattered single wildtype rat hepatocytes by day 10 post-implantation and clusters of wildtype rat hepatocytes by day 50 (NTBC cycling applied between day 10 and day 50 post-implantation).

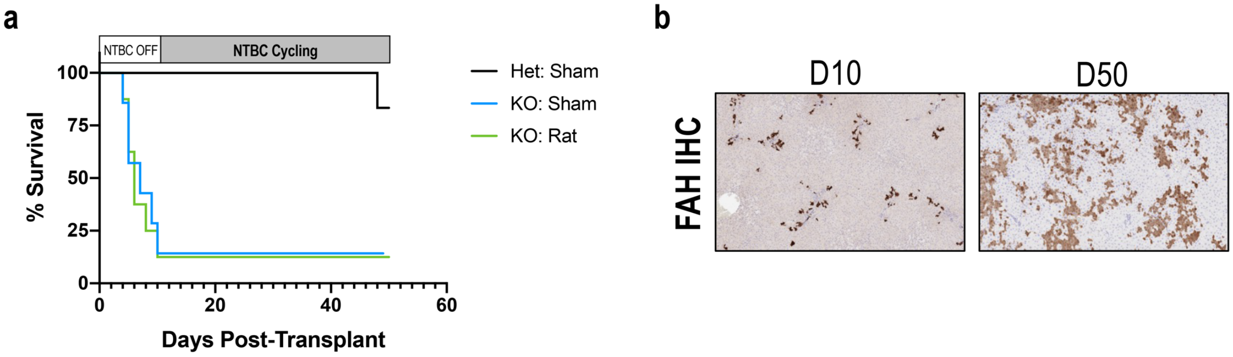

Supplementary Figure 2. Engraftment of Mouse and Human Donor Hepatocytes in FRG Rats

A. Serum mouse albumin levels in 3 individual murine-hepatocyte engrafted FRG rats versus serum from 2 non-engrafted FRG rats over time. Open boxes indicate when animals were euthanized for experimental purposes. **B.** Repopulation rates of mouse hepatocytes in FRG rats shown as the ratio of +FAH staining to total area of the liver lobule (average of 3 liver lobes, from 2 FRG rats). Representative images from 1 high-engrafted and 1 low-engrafted rat are shown. **C.** Time-course of human albumin in the serum of FRG rats engrafted with freshly-thawed, non-modified human hepatocytes (donor FCL). Each line represents an individual animal over time. Pink and blue dots represent female and male recipient rats, respectively. Open circles represent animals euthanized for experimental purposes; solid circles at endpoints show animals that did not survive. **D.** Correlation of serum human albumin levels with human hepatocyte repopulation rate (each dot represents the average of 2 liver lobes from the same FRG rat). Representative images from the highest and lowest engrafted rats are shown to the right. **E.** FAH IHC showing efficient engraftment of different human hepatocyte donors (FCL, EYP, PXB), either immediately upon thawing cryopreserved hepatocytes, or after *in vitro* culturing for 3-10 days.

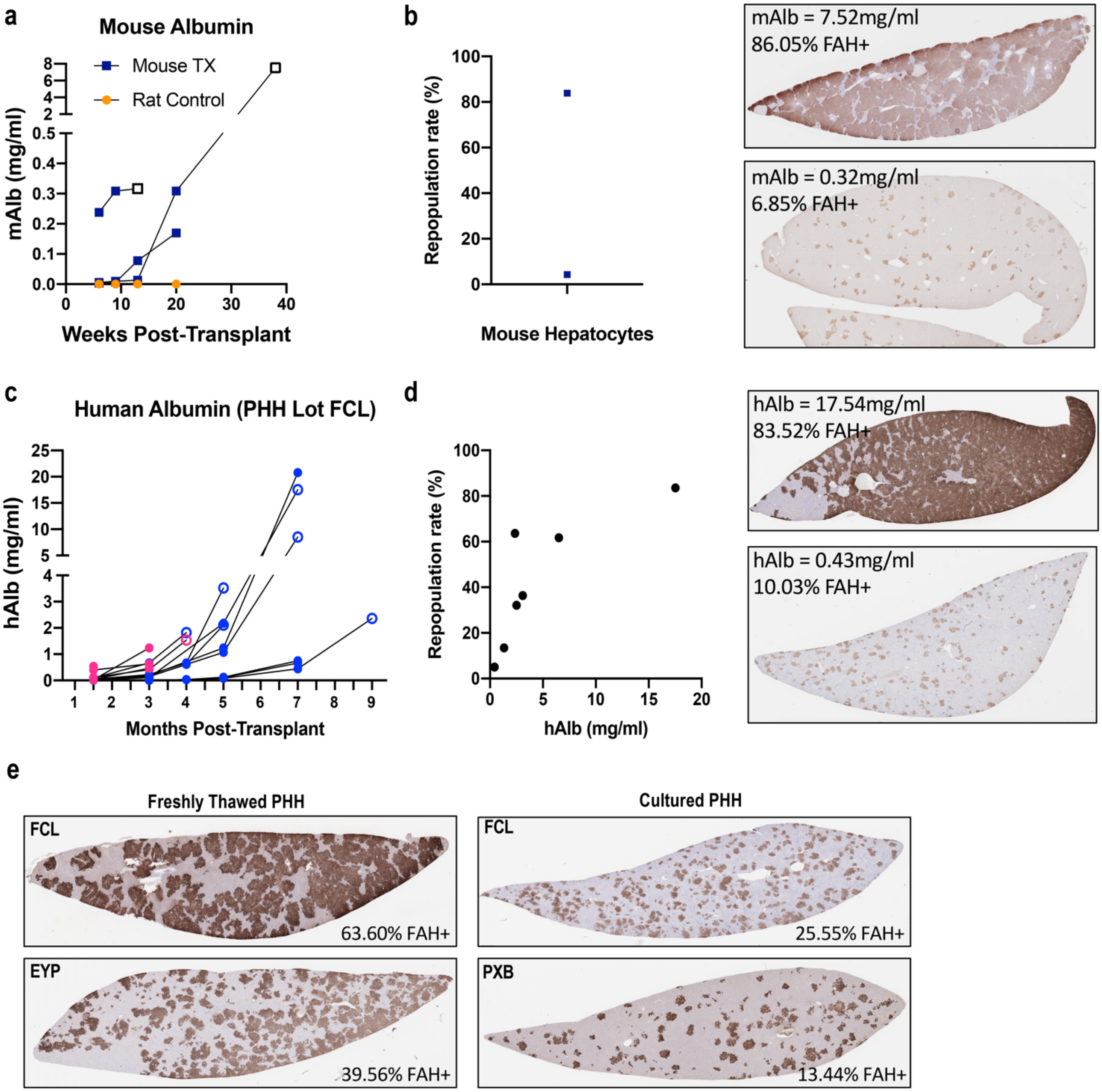
a
Mouse Albumin
Mouse TX
Rat Control
mAlb (mg/ml)
Weeks Post-Transplant
b
Repopulation rate (%)
Mouse Hepatocytes
mAlb = 7.52mg/ml
86.05% FAH+
mAlb = 0.32mg/ml
6.85% FAH+
c
Human Albumin (PHH Lot FCL)
hAlb (mg/ml)
Months Post-Transplant
d
Repopulation rate (%)
hAlb (mg/ml)
hAlb = 17.54mg/ml
83.52% FAH+
hAlb = 0.43mg/ml
10.03% FAH+
e
Freshly Thawed PHH
Cultured PHH
FCL
63.60% FAH+
FCL
25.55% FAH+
EYP
39.56% FAH+
PXB
13.44% FAH+

Supplementary Figure 3. Engraftment of Ex-vivo Modified Human Hepatocytes

A. Serum human albumin levels in FRG rats engrafted with non-modified, freshly-thawed human hepatocytes (donor FCL) in black, or human hepatocytes (donor FCL or EYP) infected with Lentivirus for 30 minutes in suspension before engraftment (green). **B.** Serum human albumin levels in FRG rats engrafted with non-modified, cultured human hepatocytes (donor FCL or PXB) in black, or human hepatocytes infected with Lentivirus for 1hr, following 6 days in culture, before engraftment (green). Animals with endpoints marked in red were harvested for histological examination.

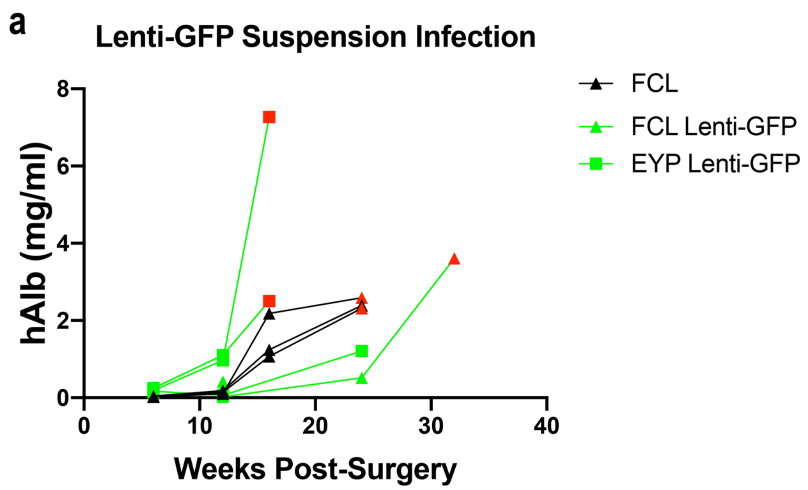

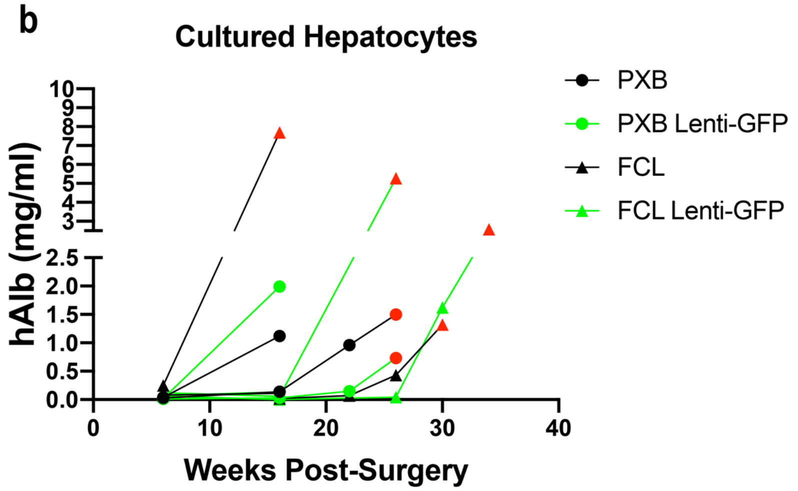

RT-PCR Probes:

Gene	Probe Sequence	Forward Primer	Reverse Primer
rFah	TGGAAATGGCTTTCTTTGTAGGCCCT	GCCAGCAAACGCTTAGACATG	GGCTCGCCGAATCTGTTC
rAlb	TCAACAAGGAGTGCTGTCACGGC	GGCAACAGACCTCACCAAA	CATCCGCGCATTCCAACAG
rAsgr1	TCGTCCAGCAACACATGGGC	TCCTGGGAGGAGCAGAGAT	GGGTCCGTTCTGGTCAGTTAG
rTtr	CCATTCCATGAATACGCAGAGGTGGT	GGAAGGCTCTTGGCATTTCC	AGGGCTGCGATGGTGTAGTG
rC3	ACGTGCTGCCCAGTTTCGAAGTC	TGCAGAGTTTGAGGTGAAGGAAT	CCAGGCCCTTTGGGTCATC
rC5	TCTTCCGGGTCGGATCATCCGA	ACGTCATTTCAGCACCCAAA	GCCATGGGCTTGAATTACGAC
rActa2	CGCCGCTGAACCCTAAGGCCAA	GAGCATCCGACCTTGCTAACG	AGCACAGCCTGAATAGCCACATAC
rDes	ACATGTCCAAGCCAGACCTCACAGC	GAACAGCAGGTCCAGGTAGA	TAGCCGCAATGGTCTCATAC
rVim	ACAAGGTGCGCTTCCTCGAGCAG	ACCGCTTCGCCAACTACATC	AGCTCGGCCAGCAGGATT
rPdgfrb	TCAAGTTCAGCTCCAGTGATGTGGG	TCGAGCACCTTTGTTCTGACAT	AGGGCACCTGGGACATCTGTT
rTimp1	CACAGGTTTCCGGTTCGCCTACA	CGACGCTGTGGGAAATGC	CGGTTCTGGGACTTGTGGACAT
rTimp2	CAATGGGCACCAGGCCAAGTTC	GACTGGGTCACAGAGAAGAGCAT	ACGCGCAAGAACCGTCACTT
rGapdh	TTTGGCATCGTGGAAGGGCTCAT	GCCAAGGTCATCCATGACAAC	GGGCCATCCACAGTCTTCTG
